# Living evidence of a fossil survival strategy raises hope for warming-affected corals

**DOI:** 10.1126/sciadv.aax2950

**Published:** 2019-10-09

**Authors:** Diego K. Kersting, Cristina Linares

**Affiliations:** 1 Working Group on Geobiology and Anthropocene Research, Institute of Geological Sciences, Freie Universität Berlin, 12249 Berlin, Germany.; 2 Departament de Biologia Evolutiva, Ecologia i Ciències Ambientals, Facultat de Biologia, Institut de Recerca de la Biodiversitat (IRBIO), Universitat de Barcelona, 08028 Barcelona, Spain.

## Abstract

Climate change is affecting reef-building corals worldwide, with little hope for recovery. However, coral fossils hint at the existence of environmental stress–triggered survival strategies unreported in extant colonial corals. We document the living evidence and long-term ecological role of such a survival strategy in which isolated polyps from coral colonies affected by warming adopt a transitory resistance phase, in turn expressing a high recovery capacity in dead colony areas. Such processes have been described in fossil corals as rejuvenescence but were previously unknown in extant reef-builder corals. Our results based on 16 years of monitoring show the significance of this process for unexpected recoveries of coral colonies severely affected by warming. These findings provide a link between rejuvenescence in fossil and extant corals and reveal that beyond adaptation and acclimatization processes, modern scleractinian corals show yet undiscovered and highly effective survival strategies that help them withstand and recover from rapid environmental changes.

## Teaser

The discovery of a fossil survival strategy in modern corals explains unexpected recoveries after warming-induced mortalities.

## INTRODUCTION

Corals are subjected to climate change–related impacts worldwide, with overwhelming evidence of mass mortalities affecting vast geographical areas in tropical (*1*) and temperate seas (*2*). Although several studies have indicated that repeated exposure to increased water temperature can influence resistance in corals (*3*–*6*), there is high uncertainty regarding potential adaptive responses (*7*), and such responses either cannot be identified by the available long-term data or might be masked by the severity of the impacts (*8*, *9*). Searching for clues in the fossil record has previously provided crucial information for understanding the range of responses of coral reefs to climate change (*10*). Survival strategies, hypothetically triggered by adverse environmental conditions, have been reported in fossil corals (*11*–*16*) but remain undiscovered and thus unstudied in extant colonial corals. Polyp contraction or rejuvenescence [after redefinition by Fedorowski (*11*)] is a frequently reported process in fossil corals (mainly Rugosa) and is defined as the action of a polyp leading to a reduction in its dimensions by leaving part of some skeletal structures in a calix outside its new external wall. Rejuvenescence and its ecological implications have never been described in modern scleractinian colonial corals, which are currently widely and intensively exposed to environmental degradation and climate change–related impacts. Gaining knowledge of the occurrence of this survival strategy and its role in population recovery will allow clarification of the resistance processes in these emblematic ecosystems in response to current and past environmental changes.

Using 16 years of monitoring data from a permanent transect, we report the discovery of rejuvenescence as a warming-driven survival strategy in a temperate zooxanthellate reef builder, the Mediterranean scleractinian *Cladocora caespitosa*. This species is considered as a relict reef builder and a missing link to both the tropical reefs and the reefal ecosystems that occurred in the Mediterranean Sea before the end of the Messinian (*17*, *18*). However, large bioconstructions by this coral, which are frequent in the fossil record (*17*), have become extremely rare in the modern Mediterranean Sea (*19*, *20*). One of the few sites still harboring large *C. caespitosa* colonies and patch reefs is located in a semi-enclosed bay in the Columbretes Islands Marine Reserve [northwestern (NW) Mediterranean Sea, Spain] (*20*). These corals have been uninterruptedly monitored since 2002, allowing quantification of the impact of recurrent warming-induced mortalities caused by a rapid polyp necrosis process without previous bleaching (*8*). The intensity and frequency of the mortalities (*8*), the slow dynamics of the species [i.e., slow growth and low recruitment rates, (*21*)], and the lack of evidence of potential acclimatization or adaptation processes (*8*, *22*) predict a discouraging scenario for *C. caespitosa*, which is classified as an endangered species on the International Union for Conservation of Nature (IUCN) Red List (*23*).

## RESULTS AND DISCUSSION

**Fig. 1:**
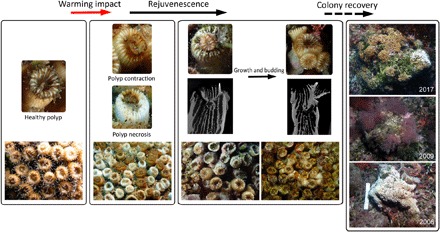
Rejuvenescence-mediated recovery at the polyp and colony levels in *Cladocora caespitosa*. Summer heat waves trigger polyp contraction and tissue necrosis. Most of the warming-affected polyps die during the necrosis events, but some survive via rejuvenescence, characterized by a drastic reduction in polyp size and the partial retreat of the polyp from the original skeletal structures (detailed information is provided in Figs. 2 and 3). Rejuvenated polyps regrow and undergo budding, eventually recolonizing dead colony areas. The right panel shows the death and recovery of a colony affected by warming in 2006 (the white color of the colony is given by the denuded coral skeleton after the death of the polyps, not by bleaching). After the necrosis event, dead colony areas are eventually overgrown by algae (2009). By 2017, the colony shows a recovery of ca. 80%, together with a recently necrosed area to the right. Scale in 2006: 25 cm (photo credit: D. K. Kersting, Freie Universität Berlin).

**Fig. 2:**
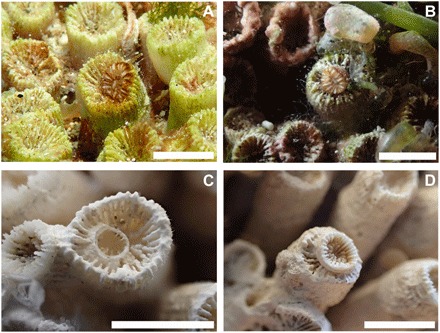
Rejuvenated *Cladocora caespitosa* polyps and related skeletal structures. (**A**) *C. caespitosa* polyp showing a drastic size reduction shortly after a necrosis event. (**B**) Rejuvenated polyp regrowing its skeleton inside a partially abandoned calix. (**C**) Calix showing the first stages of rejuvenation, with the contracted polyp retreated to the center-left portion of the calix; note how some septa show continuity inside the new external wall. (**D**) Rejuvenated calix growing over the abandoned calix. Scale bars, 0.5 cm (photo credit: D. K. Kersting, Freie Universität Berlin).

**Fig. 3:**
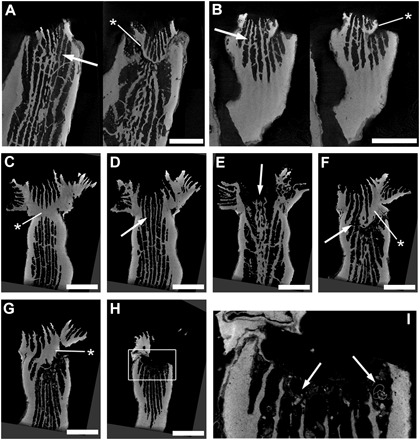
3D computed tomography sections in *C. caespitosa* calices showing rejuvenation. Septa and columnella partially connect through the abandoned and rejuvenated calices [arrows in (**A**) to (**F**)], while a new external wall is built [asterisks in (A) to (**G**)], leaving the rest of the skeletal structures and a portion of the original external wall outside the rejuvenated calix and characteristically marking the inner corallite structure. The new calix eventually recovers its original diameter, and the polyp starts budding (C to **H**). The partially abandoned calix, exposed to surrounding water, eventually fills with debris and foraminifera [arrows in (**I**), zoomed-in white rectangle of (H)]. Scale bars, 0.25 cm. Note that the sections belong to three corallites; (A) and (B) show two sections of two different corallites, while (C) to (I) belong to the same one.

**Fig. 4:**
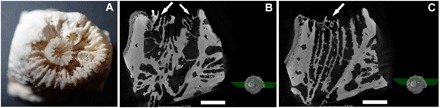
3D computed tomography sections of a *C. caespitosa* calix showing three new calices. (**A**) Photograph of the scanned corallite. (**B** and **C**) Arrows point to the new calices, which show connections to the skeletal structures of the original polyp. Scale bars: 0.15 cm (photo credit: D. K. Kersting, Freie Universität Berlin).

We found polyp rejuvenescence to be a common and effective survival strategy in *C. caespitosa* colonies affected by warming. During the first years after mortality events, no evident recovery processes were detected, with necrosed colonies remaining dead and being rapidly covered by epibionts (*8*). Colonies of *C. caespitosa* are phaceloid, with independent corallites and polyps not connected by the coenosarc; these traits make the regeneration of adjacent damaged tissue by unaffected polyps very difficult (*8*, *24*). However, yearly monitoring of the 243 individually tagged *C. caespitosa* colonies eventually revealed that 38% of the warming-affected colonies showed clear recovery signs, with living polyps covering colonies that died during previous mortality events. The first detection of these signs of recovery generally occurred several years after the last major mortality event, when recovered areas reached sizes large enough to be easily identified by visual inspection and photographs (Fig. 1 and fig. S1). A closer examination of the dead colonies revealed the occurrence of rejuvenated polyps, evidenced by the presence of a small calix inside the original external wall (Fig. 2). As described in fossil corals, the rejuvenation process in *C. caespitosa* was characterized by a drastic reduction in polyp size (Fig. 2) and its partial retreat from the inner calicular skeletal structures and the calicular rim, which are left outside of a newly formed calix and external wall while maintaining a connection to some of the old skeletal structures, as further evidenced through three-dimensional (3D) computed tomography (Fig. 3). We found that this process was always characterized by a single polyp shrinking inside the original calix. However, even if very rarely, on a few occasions, up to three new calices were found inside the original one and to share skeletal structures with it (Fig. 4). This is worth mentioning because it reveals that this process could occasionally originate from several isolated but still-living fragments of the same polyp, representing remnants from warming-induced necrosis. This last process could be similar to some recoveries associated with cryptic tissue described or hypothesized at a colony level in tropical corals (*25*–*27*), and it could shed light onto the origin and causes of similar skeletal structures (several calices inside the original one) described in fossil corals (*14*). The different stages of rejuvenated and postrejuvenated polyps found on the colonies showed that, after surviving the warming events in their rejuvenated form, *C. caespitosa* polyps eventually recover their size and grow over old, epiphyte-covered dead coral areas through budding, clearly outcompeting macroalgae (Fig. 1 and fig. S1). We disregarded a potential sexual origin (i.e., larval settlement) of the rejuvenated polyps for the following reasons. First, computed tomography scan microstructural images show that skeletal structures connect through the abandoned and rejuvenated calices. Second, *C. caespitosa* displays extremely low recruitment rates (*21*), which would hardly explain the number of rejuvenated polyps found on the colonies. Last, this species reproduces after the summer (*28*), coincident with the end of the warming-induced mortality events (when occurring); therefore, it would be impossible (in terms of timing and polyp size) for the rejuvenated polyps found during and immediately after the coral mortalities (e.g., Fig. 2A) to have resulted from recently settled larvae.

**Fig. 5:**
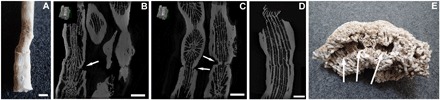
Rejuvenescence marks at the corallite and colony levels. (**A**) External rejuvenescence scar in a corallite. (**B** and **C**) 3D computed tomography sections showing rejuvenescence scars (arrows) in corallites grown many centimeters after the rejuvenescence process. (**D**) 3D computed tomography section of a rejuvenated calix in a *C. caespitosa* fossil corallite (Holocene) from Menorca (NW Mediterranean Sea). (**E**) Inner recovery interface or discontinuity (arrows) in a colony fragment. Scale bars, 0.25 cm (photo credit: D. K. Kersting, Freie Universität Berlin).

The pace and extent of recoveries were heterogeneous among colonies displaying rejuvenescence, with an annual average recovery rate of 4.27 ± 2.70% (± SD, *n* = 77; table S1). Near-full recoveries (80 to 90%, Fig. 1 and fig. S1) were observed in 13% of the rejuvenated colonies and took over 10 years, which explains why this process remained undiscovered for a long period of time (*8*). Recovered colonies can be identified only by external visual examination if their life histories are known (i.e., using long-term permanent transects) because, once recovered, the necrosed colony areas remain detectable only as a contrasted interface in the inner colony and corallite structures (Fig. 5). Bearing this in mind, it is very plausible that these mechanisms were overlooked in short-term assessments or when using nonpermanent transects, as recovered colonies may have a misleadingly healthy and undisturbed appearance after several years. However, the characteristic structures and markers (see also the fillings by debris and foraminifera in Fig. 3) left behind in the skeleton allow identification of this process and thus reconstructing information on past periods of stress, not only in extant colonies but also in fossil samples (Fig. 5).

Rejuvenescence processes have been attributed to environmental or physiological stress in fossil solitary corals (*11*–*16*). In *C. caespitosa*, the described process occurred concomitantly with the coral mortalities triggered during summer heat waves (*8*). In those summers, the corals were subjected to high physiological stress caused by high temperatures (*29*), while energetic constraints resulted from warming-enhanced water stratification (*30*). During these periods of stress, the polyp’s metabolic activity would have been reduced, and the polyp would have decreased in size and partially abandoned its skeleton, thus reducing CaCO_3_ secretion rates and the whole-animal energy demand, as previously hypothesized in fossil corals (*13*). This would allow for less energy consumption in such an energy-limited environment, especially true in the case of the energetically expensive process of calcification, resulting in a trade-off between growth/calcification costs and survival. In contrast, as described above, even polyps already severely affected by necrosis would be able to trigger these strategies from remnant tissue fragments. Altogether, rejuvenated *C. caespitosa* polyps would gain a greater chance of withstanding the temporarily unfavorable conditions by adopting a sort of transitory resistant form, representing an exceptional example of a rapid response or survival strategy of corals facing global warming.

We show that beyond potential adaptation or acclimatization processes, modern scleractinian corals harbor mechanisms allowing them to survive through transitory environmental degradation linked to those found in extinct Paleozoic Rugosa corals. Our findings provide the first insight into this kind of process in extant corals to better understand and interpret its occurrence and triggers in the fossil record, while the characteristic skeletal structures described here will serve as stress markers in extant and fossil colonies. As we show, these strategies may allow corals to withstand and effectively recover from warming-triggered mortality periods, making them more resilient than previously thought. However, some doubts arise related to the effectiveness of such strategies in a scenario of increased frequency of thermal anomalies (*31*), which may outpace the recovery capacity of long-lived corals (*1*). Nevertheless, the existence of this or other similar survival strategies is a narrow window of opportunity for corals to deal with global warming.

## METHODS

### Study site

The Columbretes Islands (NW Mediterranean Sea) are located 30 nautical miles off the nearest coast (Castelló, Spain). A marine reserve encircles the archipelago, covering an area of 5500 hectares. Illa Grossa (39°53.825′N, 0°41.214′E), the largest of the islets in the Columbretes, is a C-shaped, drowned, Quaternary volcanic caldera. The monitored *C. caespitosa* population is found within Illa Grossa Bay (*20*).

### Coral monitoring

A permanent transect of 243 *C. caespitosa* colonies was annually monitored in the Columbretes Islands from 2002 to 2017. Each colony was examined after the summer, when mortality-triggering thermal anomalies occur, and the extent of the necrosed surface was recorded with photographs and sketches (*8*). Monitoring of the permanent coral transect allowed not only the quantification of necrosis (*8*) but also the accurate assessment and quantification of the short- and long-term evolution of each colony after the necrosis events. In summary, this methodology has allowed us to obtain detailed (annual resolution) quantitative information on the life histories of 243 *C. caespitosa* colonies over 16 years.

Close visual inspection of necrosed colony areas, together with macrophotography, was performed to identify surviving polyps. Additional macrophotographs of rejuvenated polyps on dead, warming-affected colonies, previously cleaned of remaining organic matter using H_2_O_2_ were taken in the laboratory.

### Recovery rates

Average annual recovery rates through rejuvenescence and posterior asexual reproduction were calculated considering the colony surface (%, recovered versus necrosed area) recovered from the last major mortality event suffered by each colony to the last annual monitoring.

### 3D computed tomography

Samples of rejuvenated corallites (*n* = 21) from the Columbretes Islands, previously cleared of organic matter, and a corallite sample from a Holocene fossil reef in Menorca (Balearic Islands, Western Mediterranean Sea) (*32*) were subjected to microtomographic analysis at the Museum für Naturkunde Berlin, using a phoenix nanotom x-ray tube at 100 kV and 135 μA. Scan images of longitudinal corallite sections were used to identify and describe the polyp reduction processes.

## Supplementary Material

http://advances.sciencemag.org/cgi/content/full/5/10/eaax2950/DC1

Download PDF

Living evidence of a fosil survival strategy raises hope for warming-affected corals

## SUPPLEMENTARY MATERIALS

Supplementary material for this article is available at http://advances.sciencemag.org/cgi/content/full/5/10/eaax2950/DC1 Fig. S1. Long-term rejuvenescence-mediated recoveries of warming-affected *C. caespitosa* colonies. Table S1. Recovery data and annual recovery rates in transect colonies showing rejuvenation processes.
