# Supplementary material for: Living evidence of a fossil survival strategy raises hope for warming-affected corals
Source: Sci Adv. 2019 Oct 9;5(10):eaax2950. doi: 10.1126/sciadv.aax2950 (PMC6785258; doi:10.1126/sciadv.aax2950)
Supplement: http://advances.sciencemag.org/cgi/content/full/5/10/eaax2950/DC1 [file supp_5_10_eaax2950__index.html]

Science Advances | Science AdvancesAAASSearchScience AdvancesMenu

## Supplementary Materials

**This PDF file includes:**

- Fig. S1. Long-term rejuvenescence-mediated recoveries of warming-affected *C. caespitosa* colonies.
- Table S1. Recovery data and annual recovery rates in transect colonies showing rejuvenation processes.

Download PDF

**Files in this Data Supplement:**

- Adobe PDF - aax2950\_SM.pdf
