# Supplementary material for: Living evidence of a fossil survival strategy raises hope for warming-affected corals
Source: Sci Adv. 2019 Oct 9;5(10):eaax2950. doi: 10.1126/sciadv.aax2950 (PMC6785258; doi:10.1126/sciadv.aax2950)
Supplement: Download PDF [file aax2950_SM.pdf]

Science Advances
AAAS

advances.sciencemag.org/cgi/content/full/5/10/eaax2950/DC1

Supplementary Materials for

Living evidence of a fossil survival strategy raises hope for warming-affected corals

Diego K. Kersting* and Cristina Linares

*Corresponding author. Email: diegokersting@gmail.com

Published 9 October 2019, *Sci. Adv.* **5**, eaax2950 (2019)
DOI: 10.1126/sciadv.aax2950

This PDF file includes:

Fig. S1. Long-term rejuvenescence-mediated recoveries of warming-affected *C. caespitosa* colonies.
Table S1. Recovery data and annual recovery rates in transect colonies showing rejuvenation processes.

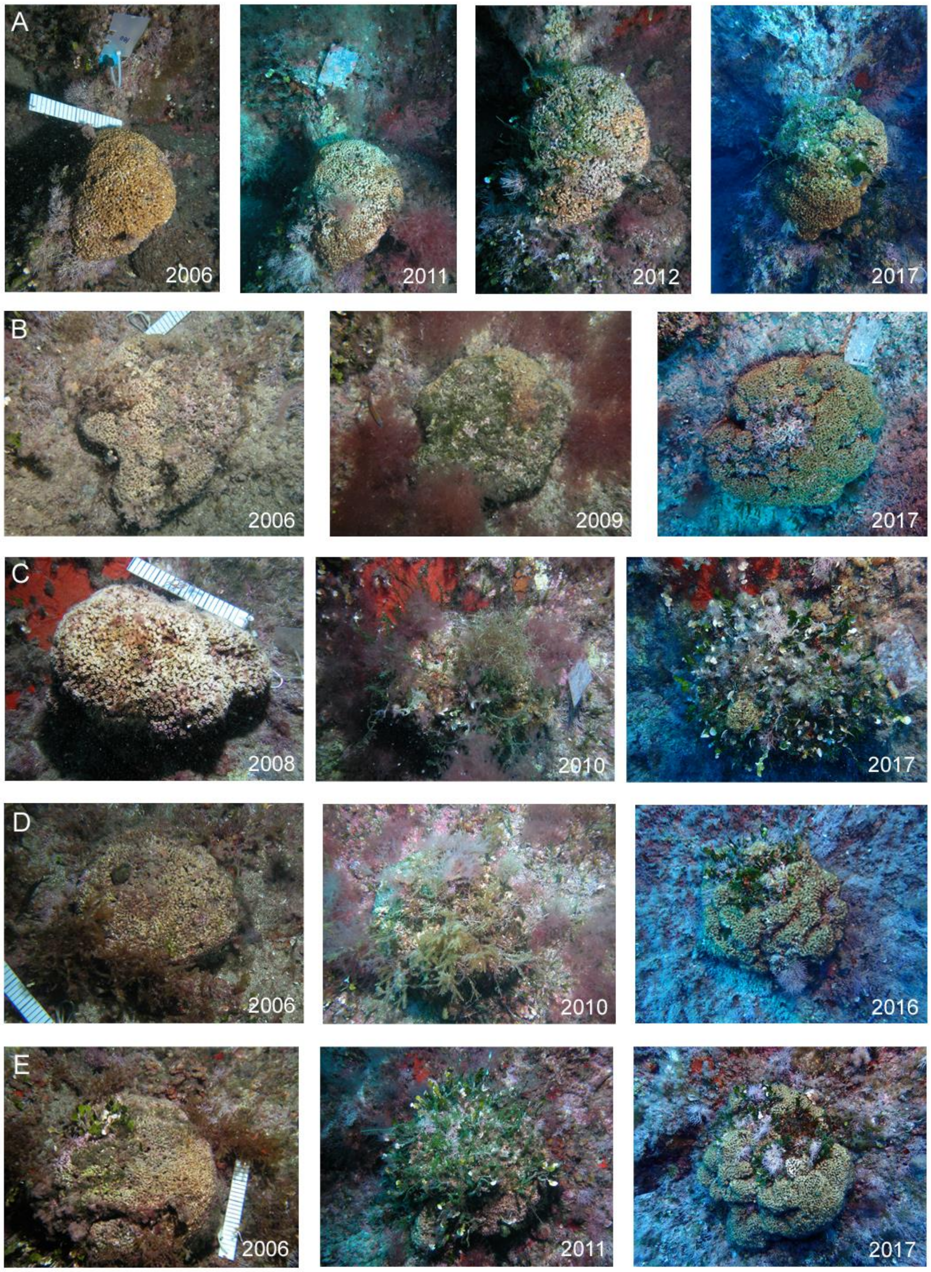

Fig. S1. Long-term rejuvenescence-mediated recoveries of warming-affected *C. caespitosa* colonies. (A) Colony that suffered necrosis in 2011 and showed a 50% recovery in 2017. (B) Necrosis suffered in 2006, with an 80% recovery in 2017. (C) Example of low recovery. Necrosis suffered in 2008, with a 10% recovery in 2017. (D) Necrosis suffered in 2006, with a 90% recovery in 2016. (E) Necrosis suffered in 2006, with an 80% recovery in 2017. Note that algal overgrowth in necrosed colony areas is generalized at the intermediate recovery stages, but algae are eventually outcompeted by the coral once it has successfully recovered. Scale: 25 cm. (Photo Credit: D. K. Kersting, Freie Universität Berlin).

Table S1. Recovery data and annual recovery rates in transect colonies showing rejuvenation processes.

*Last monitoring year is in general terms 2017, however in some colonies it might be earlier due to whole colony breakages after storm events.

Colony	Year of major necrosis event	Total recovery (%)	No. years until last monitoring*	Annual recovery rate (%)
b3_100	2011	50	6	8.33
b3_14	2006	30	11	2.73
b3_17	2006	40	12	3.33
b3_28	2009	30	8	3.75
b3_37	2015	10	2	5.00
b3_43	2006	30	10	3.00
b3_44	2006	10	7	1.43
b3_45	2004	20	13	1.54
b3_5	2006	30	11	2.73
b3_50	2003	30	14	2.14
b3_50'	2006	10	10	1.00
b3_51	2012	20	5	4.00
b3_52	2006	30	11	2.73
b3_56	2003	30	14	2.14
b3_57	2006	80	11	7.27
b3_6	2012	30	5	6.00
b3_62	2015	10	2	5.00
b3_64''	2006	10	8	1.25
b3_65	2004	60	13	4.62

b3_66	2006	10	8	1.25
b3_67	2006	10	6	1.67
b3_69	2006	50	10	5.00
b3_7	2006	80	11	7.27
b3_70	2006	50	10	5.00
b3_70'	2010	30	2	15.00
b3_70''	2006	10	8	1.25
b3_73	2006	30	11	2.73
b3_75	2004	60	13	4.62
b3_76	2006	90	11	8.18
b3_77	2006	70	11	6.36
b3_78	2008	90	9	10.00
b3_79	2006	70	12	5.83
b3_80	2003	50	14	3.57
b3_81	2006	30	11	2.73
b3_82	2006	60	11	5.45
b3_83	2006	50	9	5.56
b3_85	2006	70	10	7.00
b3_87	2006	80	11	7.27
b3_9	2006	50	11	4.55
b3_93	2006	80	11	7.27
b3_97	2006	20	11	1.82
b3_99	2004	40	13	3.08
b8_10	2006	20	11	1.82
b8_11	2006	90	11	8.18
b8_13	2006	60	11	5.45

b8_16	2004	30	13	2.31
b8_18	2004	30	13	2.31
b8_19	2004	20	13	1.54
b8_20	2006	60	11	5.45
b8_23'	2004	20	13	1.54
b8_26	2003	30	14	2.14
b8_34	2006	30	11	2.73
b8_39	2006	70	11	6.36
b8_40	2008	50	9	5.56
b8_46'	2015	10	2	5.00
b8_46	2003	60	14	4.29
b8_47'	2015	20	2	10.00
b8_5	2006	30	11	2.73
b8_56	2015	20	2	10.00
b8_57	2003	10	14	0.71
b8_59	2006	30	6	5.00
b8_6	2003	30	14	2.14
b8_64	2008	40	9	4.44
b8_65	2006	40	11	3.64
b8_66	2005	30	12	2.50
b8_72	2009	30	8	3.75
b8_77	2004	10	13	0.77
b8_78	2006	20	11	1.82
b8_8	2011	10	6	1.67
b8_85	2006	30	11	2.73
b8_86	2006	30	11	2.73

b8_87	2004	40	13	3.08
b8_89	2006	90	10	9.00
b8_9	2005	40	12	3.33
b8_90'		80	11	7.27
b8_94	2003	80	14	5.71
b8_99'	2006	10	10	1.00
